# Thalamic Atrophy Without Whole Brain Atrophy Is Associated With Absence of 2-Year NEDA in Multiple Sclerosis

**DOI:** 10.3389/fneur.2019.00459

**Published:** 2019-05-03

**Authors:** Katariina Hänninen, Matias Viitala, Teemu Paavilainen, Jari O. Karhu, Juha Rinne, Juha Koikkalainen, Jyrki Lötjönen, Merja Soilu-Hänninen

**Affiliations:** ^1^Division of Clinical Neurosciences, Turku University Hospital, University of Turku, Turku, Finland; ^2^Department of Mathematics and Statistics, University of Turku, Turku, Finland; ^3^Medical Imaging Centre of Southwest Finland, Turku, Finland; ^4^Turku PET Centre, University of Turku, Turku, Finland; ^5^Combinostics Ltd., Tampere, Finland

**Keywords:** multiple sclerosis, magnetic resonance imaging, brain atrophy, NEDA, thalamus, disability, cognition

## Abstract

**Purpose:** To study which brain volume measures best differentiate early relapsing MS (RMS) and secondary progressive MS (SPMS) patients and correlate with disability and cognition. To test whether isolated thalamic atrophy at study baseline correlates with NEDA (no evidence of disease activity) at 2 years.

**Methods:** Total and regional brain volumes were measured from 24 newly diagnosed RMS patients 6 months after initiation of therapy and 2 years thereafter, and in 36 SPMS patients. Volumes were measured by SIENAX and cNeuro. The patients were divided into subgroups based on whole brain parenchyma (BP) and thalamic atrophy at baseline. Standard scores (*z*-scores) were computed by comparing individual brain volumes against healthy controls. A *z*-score cut-off of −1.96 was applied to separate atrophic from normal brain volumes. The Expanded Disability Status Scale (EDSS) and Symbol Digit Modalities Test (SDMT) were assessed at baseline and at 2 years. Differences in achieving NEDA-3, NEDA-4, EDSS progression, and SDMT change were analyzed between patients with no thalamic or BP atrophy and in patients with isolated thalamic atrophy at baseline.

**Results:** At baseline, 7 SPMS and 12 RMS patients had no brain atrophy, 8 SPMS and 10 RMS patients had isolated thalamic atrophy and 2 RMS and 20 SPMS patients had both BP and thalamic atrophy. NEDA-3 was reached in 11/19 patients with no brain atrophy but only in 2/16 patients with isolated thalamic atrophy (*p* = 0.012). NEDA-4 was reached in 7/19 patients with no brain atrophy and in 1/16 of the patients with isolated thalamic atrophy (*p* = 0.047). At 2 years, EDSS was same or better in 16/19 patients with no brain atrophy but only in 5/17 patients with isolated thalamic atrophy (*p* = 0.002). There was no significant difference in the EDSS, relapses or SDMT between patients with isolated thalamic atrophy and no atrophy at baseline.

**Conclusion:** Patients with isolated thalamic atrophy were at a higher risk for not reaching 2-year NEDA-3 and for EDSS increase than patients with no identified brain atrophy. The groups were clinically indistinguishable. A single measurement of thalamic and whole brain atrophy could help identify patients needing most effective therapies from early on.

## Introduction

The quantification of brain atrophy by MRI has become an increasingly important part of evaluating neurodegeneration in MS (1, 2). Atrophy measures can reflect the damage on the central nervous system (CNS) caused by the pathological processes of the disease. However, some contributors to volumetric change, such as fluid shifts, are potentially more reversible than others, e.g., axonal and/or neuronal loss. Brain atrophy occurs in all clinical stages of untreated MS patients at a rate of 0.5–1.35%/year, in comparison with 0.1–0.3%/year in healthy individuals (1). Thalamus and other deep gray matter (GM) nuclei are among the first GM structures to be affected in MS. Several studies have shown associations between GM atrophy and measures of disease progression, such as accumulation of physical disability (3, 4) and cognitive impairment (5). Thalamic atrophy occurs from early on in the disease course (6) and it has been associated with the transition from clinically isolated syndrome (CIS) to definite MS (7). Thalamic atrophy has been shown to correlate with accumulation of disability in patients with MS (8).

Brain atrophy measures could help predict the risk of future cognitive impairment and disability progression. Knowledge of the extent of brain atrophy could affect early treatment decisions, such as choice of disease modifying therapy (DMT). However, brain volume is not routinely measured in normal clinical practice due to several reasons. There are no standardized guidelines on how to measure brain atrophy in MS and the measures are potentially influenced by technical, biological, and pharmacological factors (2). SIENA and SIENAX are the most established automated methods for measuring brain atrophy. Even though these methods are automated, their use in image analysis requires time consuming refinement of the images. In recent years, several other automated methods of brain segmentation, such as the FIRST tool from the FMRIB Software Library (FSL) (9) and FreeSurfer, have been introduced (10). These methods have mainly been used in research setting and their applicability in real life and implementation in evaluating treatment outcomes is not yet included in the guidelines for the use of MRI in MS (2).

NEDA (no evidence of disease activity) has emerged as a potential treatment target in patients with relapsing MS (RMS). NEDA-3 is determined by no clinical relapse, no confirmed Expanded Disability Status Scale (EDSS) disability progression sustained during the follow-up period, no new Gadolinium (Gd)-enhancing lesions, and no new or enlarging T2 lesions in MRI and NEDA-4 by annualized whole brain volume loss ≤ 0.4% as a fourth variable (11). NEDA-3 has been used to evaluate DMT (12, 13) and is becoming integrated into clinical decision-making. It has been shown to predict long term disability (11). However, the implementation of NEDA-4 in clinical practice is currently hindered by logistical and technical difficulties (11).

A recent study showed, that MRI-based brain volumetry at a single time point was able to reliably distinguish MS patients with isolated thalamus atrophy from those without brain atrophy (14). These patients could not be clinically distinguished from the RMS patients with no thalamic or whole brain atrophy, but patients with thalamus and BP atrophy showed significantly higher EDSS scores than patients in the other groups. The authors suggested that grouping patients based on MRI-based volumetry could provide information that goes beyond clinical assessments and could help identify MS patients at risk of developing widespread atrophy and disease progression.

We performed a prospective 2-year clinical follow up study in a single academic center. A fully automated multi-atlas segmentation based method, cNeuro (15), was used for the regional brain volume assessment and SIENAX for measurement of whole brain volume and SIENA for volume changes. Our first objective was to study which brain volume measures best differentiate early RMS and secondary progressive MS (SPMS) patients and correlate with disability and cognition. Secondly, we wished to test whether isolated thalamic atrophy at study baseline correlates with clinical disability worsening, relapses, cognition, and MRI outcomes at 2 years. To meet these aims, total and regional brain volumes were measured from 3D T1 brain MR images of 24 newly diagnosed RMS patients and 36 SPMS patients at baseline and after 2 years. The correlation of global and regional brain volumes with cognition and disability were analyzed at baseline and after 2 years. Secondly, the patients were divided into subgroups according to baseline thalamic and whole brain atrophy and subgroup differences in reaching NEDA-3 and NEDA-4 status, in EDSS progression and in cognitive performance after 2 years were analyzed. We have earlier shown that vitamin D supplement use is associated with less brain atrophy progression in a pooled analysis of the patients from the FREEDOMS trials (16), while others have shown that serum 25 (OH)D levels are associated with clinical and MRI outcomes in MS-patients receiving interferon-beta (17). Therefore, we included measurement of 25(OH)D levels in the baseline characteristics.

## Materials and Methods

### Ethics Committee Approval

The study was approved by the Ethics committee of Turku University Hospital and University of Turku at 21.1.2014 and written informed consent was obtained from all patients participating in the study.

### Subjects

In this prospective study, 24 patients with newly diagnosed RMS on first-line immunomodulatory treatment initiated 6 months before study baseline, and 36 patients having SPMS were included. Inclusion criteria for the RMS patients were RMS fulfilling McDonald 2010 criteria and EDSS 0-3.5 and either glatiramer acetate of interferon-beta treatment initiated within 12 months as the first DMT. In the SPMS group, patients who had entered secondary progressive stage of MS, as defined by the treating neurologist, and EDSS 4-6.5, were included. All the participants were patients at the out-patient clinic of Turku University Hospital. Exclusion criteria were malignancy, contraindications to MRI, pregnancy or planning of a pregnancy, and failure to obtain informed consent from the patient. The following background data were collected using structured StellarQ MS registry (www.stellarq.com): age, sex, date of MS diagnosis, first symptoms, neurological status findings, and EDSS at baseline and at 2 years, serum concentration of 25-OH(D) (25-hydroxyvitamin D), socioeconomic status, data on immunomodulatory drug usage, data on relapses from the date of first symptoms until the end of the study. First patient enrolled in March 2014 and last patient in January 2015. No evidence of disease activity status, NEDA-3 was determined by no relapses, no new or enhancing lesions on MRI and no disability progression from the study baseline 6 months to 2 years later. NEDA-4 was determined by NEDA-3 and annualized whole brain volume loss ≤ 0.4% were determined by SIENA.

### MRI Acquisition

MRIs were obtained from the 24 newly diagnosed RMS patients 6 months after initiating DMT and 2 years later, and from the 36 SPMS patients at study baseline and 2 years later. Images were analyzed by the same neuro radiologist with long experience in MS image analyses (JOK). Two female patients did not undergo the 2-year MRI analysis because of refusal in one and a total atrioventricular block necessitating pacemaker application before the second MRI in the other. The patients had to be clinically stable with no corticosteroid administration within 30 days before the MRI. All of the brain scans were acquired at the same radiological facility using the same scanner and same acquisition protocol settings were used for all of the scans. We aimed to minimize the pseudo atrophy effect in the RMS group by timing the baseline MRI 6 months after the onset of the DMT. It is a routine clinical practice in Finland to rescan all the patients at 6 months after initiation of DMT treatment (18).

The magnetic resonance images were obtained using a Siemens Skyra 3.0 Tesla scanner. MRI examinations included the acquisitions of high resolution pre- and post-contrast 3D T1-weighted sequence and a 3D Fluid Attenuated Inversion Recovery (FLAIR) sequence. T1-weighted images were acquired using a 3D magnetization-prepared rapid gradient-echo sequence (MPRAGE) in sagittal planes (repetition time (TR) = 2300 ms; echo time (TE) = 2.29 ms; inversion time (TI) = 900 ms; flip Angle = 8°). FLAIR-images were acquired in sagittal planes (TR = 5,000 ms; TE = 396 ms; TI = 1,800 ms; flip Angle = 120°).

### Global and Regional Brain Volume Measurement

Baseline white and gray matter volumes, normalized for subject head size, were determined by SIENAX and volume change between baseline and 2 years by SIENA on the 3D T1 MRI images prior to Gadolinium administration (19, 20) by an experienced neuro radiologist (TP). Manually generated lesion masks and the lesion-filling tool (part of FSL) were used to minimize the impact of hypo intense T1 lesions on volume measurements (21). Briefly, the lesion-filling tool uses the co-registered lesion masks and structural images (i.e., images to be filled) to fill the lesions with intensities that are similar to those in the non-lesional neighboring white matter.

In the SIENAX analyses, the same BET (Brain Extraction Tool) parameters (*f* = 0.1; *g* = 0; option “B”) were used for all images as described by Popescu et al. (22). Quality control was performed to exclude imaging artifacts and when necessary, BET parameters were further refined individually after manual correction of the segmentations.

Regional brain volumes were determined at both time points by a fully automated multi-atlas segmentation tool cNeuro (Combinostics Ltd, Tampere, Finland) from the 3D T1 MRI images that were obtained prior to Gadolinium administration (15, 23). Volumes normalized for age, sex and head size were used in the statistical analyses. The cNeuro method was used since it is a fully automated tool for brain atrophy measurement, it has a CE marking and is in clinical use at the Turku University Hospital. The segmentation method described by Koikkalainen et al. (23), and Wang et al. (24), was used to compute volumes of white-matter lesions from 3D FLAIR images.

### Clinical and Neuropsychological Assessment

The Symbol digit modalities test [SDMT (25)] was performed at baseline and at 24 months by the study nurse and the results were collected using SQ-MS. EDSS was performed by a trained neurologist with 21 years of experience in EDSS evaluation (M S-H). EDSS and SDMT evaluations were performed within 2 weeks of the MRI acquisition.

### 25(OH)D Measurement

Plasma levels of 25(OH)D were determined in Turku University Hospital clinical chemistry laboratory (Tykslab) using a chemiluminescence binding assay (Roche Diagnostics GmbH, Mannheim, Germany). The values were adjusted by the season of sampling using the following formula: –sin(2πX/ 12)–cos(2πX/12), where X is month of sample collection (26). Values adjusted for the season were used for the statistical analyses.

### Standard Score (*z*-Score) Calculation and Grouping of MS-Patients According to Atrophy Measures

Standard scores (*z*-scores) were defined by comparing individual brain volumes with corresponding volumes from the Open Access Series of Imaging Studies (OASIS) cohort of 295 healthy controls acquired using Siemens 3T scanners (27). A *z*-score cut-off of −1.96 was applied to separate pathologically atrophic from normal brain volumes for thalamus and whole brain parenchymal (BP) volume (accepting a 2.5% error probability). Based on *z*-scores the patients were divided into groups with no brain atrophy, isolated thalamic atrophy, both thalamic and whole BP atrophy, and whole BP atrophy without thalamic atrophy.

### Statistical Analysis

Cognition, disability, and brain volumes were compared between RRMS and SPMS patients. Correlation of volumes between cognition and disability were analyzed. In the group comparisons, Fisher's exact test was used for categorical parameters and Wilcoxon Rank-Sum test for continuous variables. The Pearson Correlation Coefficients were used in the correlation analyses and the significances of correlations were obtained from the t distribution. For controlling the False Discovery Rate, Benjamini-Hochberg procedure was used as correction for multiple comparisons. After grouping of the patients based on the *z*-scores, between-group differences in NEDA-3, NEDA-4, EDSS, and SDMT were tested using Fisher's exact test and validation of *p*-values was done using the Monte Carlo simulated chi2 test. Additional analysis for 2 × 2 contingency tables was done using predictive values, logistic regression derived odds ratios and confidence intervals. R was used for all the analyses and *p*-values < 0.05 were considered significant.

NEDA-3 positive predictive value was determined by the ratio of patients with thalamus atrophy not reaching NEDA-3 among all the patients with thalamus atrophy (true positives). NEDA-3 negative predictive value was determined by the ratio of patients with no thalamic atrophy reaching NEDA-3 among all patients with no thalamic atrophy (true negatives). Similarly, EDSS positive predictive value was determined by the ratio of patients with thalamus atrophy and EDSS progression among all patients with thalamus atrophy (true positives). EDSS negative predictive value was calculated with the ratio of patients with no thalamus atrophy and no EDSS progression among patients with no thalamus atrophy (true negatives). The number of patients reaching NEDA-4 was too small for a meaningful predictive value analysis.

## Results

### Baseline Clinical Characteristics and SDMT Test Results in the RMS and SPMS Groups

The baseline clinical characteristics and SDMT test results in the RMS and SPMS patients are shown in Table 1. There were significant differences between RMS and SPMS patients in age, disability, disease activity and cognition. The SPMS patients were older, had longer disease duration, higher EDSS points and lower SDMT scores. There were no significant differences between the RMS and SPMS patients in their comorbidities, alcohol consumption, education status, smoking habits, serum vitamin D levels or BMI. All patients in the RMS group started either glatiramer acetate (GA) or interferon-beta (IFNB) therapy 6 months prior to the baseline MRI. A total of 30% of the SPMS patients included in the study were using DMT's. The DMT's used by the patients are shown in Supplemental Table 1. As a local treatment practice, all patients were supplemented with vitamin D3. The mean dose of vitamin D3 supplement was 65.9 μg (range 50–150) in the RMS patients and 55.2 μg (range 10–100) in the SPMS patients. One of the patients in the SPMS group had ulcerous colitis and azathioprine medication for it. Other comorbidities included depression, arterial hypertension, glaucoma, irritable bowel syndrome, hypothyroidism, hypercholesterolemia, epilepsy, mitral prolapse, and frozen shoulder with no clustering of any comorbidities in either patient group (data not shown).

**Table 1:** Baseline demographic and clinical characteristics of the MS patients included in the study.

**Variable**	**RRMS**	**SPMS**	***p*-value**
*N*	24	36	
Age, years (mean, SD)	36.3 (7.53)	52.8 (7.28)	**<0.001**
Females/males	20/4	21/15	0.069
Disease duration, years (mean, range)	0.8 (0.7–1.6)	19.7 (5.4–35.7)	**<0.001**
EDSS (median, range)	1.2 (0.0–4.0)	4.5 (2.0–6.5)	**<0.001**
Education, years (mean, SD)	12.5 (2.30)	12.5 (2.91)	0.825
SDMT (mean, SD)	49.5 (11.82)	36.3 (10.72)	**<0.001**
BMI kg/m2 (mean, range)	26.7 (18.4–41.0)	25.3 (16.1–36.3)	0.417
Smoking, yes/no	11/13	14/22	0.662
25(OH)D, nmol/l (mean, range)	122.8 (59.7–271.6)	108.4 (25.7–268.7)	0.248
Number of relapses (mean, range)	0.7 (0.0–3.0)	0.1 (0.0–2.0)	**0.005**
Percentage of patients with DMT's	100	31	**<0.001**

*The bold values indicate statistically significant p-values*.

### MRI Characteristics in the RMS and SPMS Groups

First, we examined the differences in MRI characteristics between the RMS and SPMS patients. Significant differences between RMS and SPMS patients at both study baseline and at 2 years were detected in the white matter lesion volume, total BP, total gray and white matter, thalamus, hippocampus, cerebellar white matter, and putamen volumes. The volumes normalized for age, sex and head size were used in the analyses. The total and regional brain volumes of the patients at study baseline and at 2 years are shown in Table 2. The most significant differences between RMS and SPMS patients were found in thalamic volume (*p* < 0.001) and cerebellar WM volume (*p* < 0.001) at both time points. The numbers of T2 lesions and Gadolinium-enhancing lesions at study baseline and at 2 years in the RMS and SPMS patient groups are shown in Supplemental Table 2. We aimed to minimize the effect of pseudo atrophy in the RMS group by timing the baseline MRI 6 months after the onset of the DMT.

**Table 2:** Brain MRI findings in the RMS and SPMS patients at study baseline and at 2 years.

	**RRMS**		**SPMS**		***p*****-value**	
**Variable**	**Baseline**	**Follow-up**	**Baseline**	**Follow-up**	**Baseline**	**Follow-up**
*N*	24	23	36	35		
Gd+ lesions (*N*, %)	4/24 (17)	1/23 (4)	2/36 (6)	4/35 (11)	0.248	0.677
Total BP vol. (ml, SD)	1509.5 (109.26)	1482.3 (110.87)	1397.9 (107.11)	1404.7 (107.49)	**0.001**	**0.012**
Total WM vol. (ml, SD)	688.6 (51.40)	678.0 (45.09)	648.1 (49.05)	652.9 (53.10)	**0.011**	0.084
Total GM vol. (ml, SD)	820.7 (67.03)	804.1 (74.16)	749.9 (66.24)	751.7 (67.66)	**<0.001**	**0.007**
WM lesion vol. (ml, SD)	11.0 (8.35)	10.2 (8.43)	20.2 (12.26)	20.4 (13.29)	**<0.001**	**<0.001**
Putamen vol. (ml, SD)	4.4 (0.58)	4.4 (0.61)	4.0 (0.63)	4.0 (0.67)	**0.020**	0.065
Hippocampus vol. (ml, SD)	3.9 (0.48)	3.9 (0.55)	3.5 (0.44)	3.4 (0.48)	**0.008**	**0.006**
Thalamus vol. (ml, SD)	7.2 (0.80)	7.3 (0.82)	6.2 (0.95)	6.2 (0.97)	**<0.001**	**<0.001**
Nucleus caudatus vol. (ml, SD)	2.9 (0.41)	2.8 (0.45)	2.5 (0.40)	2.5 (0.43)	**<0.001**	**0.014**
Globus pallidus vol. (ml, SD)	1.3 (0.15)	1.2 (0.16)	1.1 (0.14)	1.1 (0.14)	**0.003**	**0.020**
Cerebellum WM vol. (ml, SD)	15.7 (2.36)	15.8 (2.25)	11.9 (2.97)	11.6 (2.62)	**<0.001**	**<0.001**
Cerebellum GM vol. (ml, SD)	55.1 (5.18)	57.1 (5.92)	54.7 (7.21)	54.1 (7.34)	0.769	0.127

*The bold values indicate statistically significant p-values*.

### Correlation of Total and Regional Brain Volumes and Lesion Volume With EDSS and SDMT

Results of the correlation analyses of the brain volumes with cognition and disability are presented in Table 3. In brief, better performance in SDMT-test significantly correlated with larger cerebellum white matter, hippocampus, thalamus, total gray matter, and total brain volumes at both baseline and follow-up, and with putamen volume at study baseline but not at follow-up. Higher EDSS significantly correlated with smaller cerebellum white matter, hippocampus, thalamus, putamen, total gray matter, and total brain volumes at both time points. White matter lesion volume positively correlated with EDSS (*p* = 0.045 at baseline, *p* = 0.015 at follow-up) and negatively with SDMT (*p* < 0.001 at both time points). The two-year data was compared cross-sectionally, because as a segmentation based tool, cNeuro is not ideal for measuring changes between two time points. However, we wanted to show that the group differences were reproducible at two different time points.

**Table 3:** Correlation of total and regional brain volumes with EDSS and SDMT in all the study patients at the study baseline and at 2 years.

		**Baseline**		**Follow-up**	
**Variable**	**Covariate**	**Pearson coorelation**	***p*-value**	**Pearson coorelation**	***p*-value**
EDSS	Total BP vol.	-0.387	**0.009**	−0.354	**0.016**
	Total WM vol.	-0.279	**0.045**	−0.220	0.097
	Total GM vol.	-0.423	**0.005**	−0.395	**0.009**
	WM lesion vol.	0.271	**0.045**	0.363	**0.015**
	Putamen vol.	-0.295	**0.034**	−0.295	**0.035**
	Hippocampus vol.	-0.317	**0.025**	−0.301	**0.034**
	Thalamus vol.	-0.335	**0.018**	−0.433	**0.005**
	Nucleus caudatus vol.	-0.343	**0.016**	−0.266	0.052
	Globus pallidus vol.	-0.245	0.062	−0.250	0.062
	Cerebellum WM vol.	-0.452	**0.003**	−0.549	**<0.001**
SDMT	Total BP vol.	0.364	**0.009**	0.384	**0.006**
	Total WM vol.	0.278	**0.043**	0.281	**0.043**
	Total GM vol.	0.387	**0.006**	0.401	**0.006**
	WM lesion vol.	-0.545	**<0.001**	−0.536	**<0.001**
	Putamen vol.	0.282	**0.041**	0.255	0.065
	Hippocampus vol.	0.382	**0.006**	0.332	**0.018**
	Thalamus vol.	0.418	**0.004**	0.419	**0.004**
	Nucleus caudatus vol.	0.339	**0.014**	0.250	0.067
	Globus pallidus vol	0.201	0.131	0.177	0.188
	Cerebellum WM vol.	0.478	**<0.001**	0.524	**<0.001**

*The bold values indicate statistically significant p-values*.

### Grouping of the Patients Based on Thalamic and BP Atrophy

**Figure 1:**
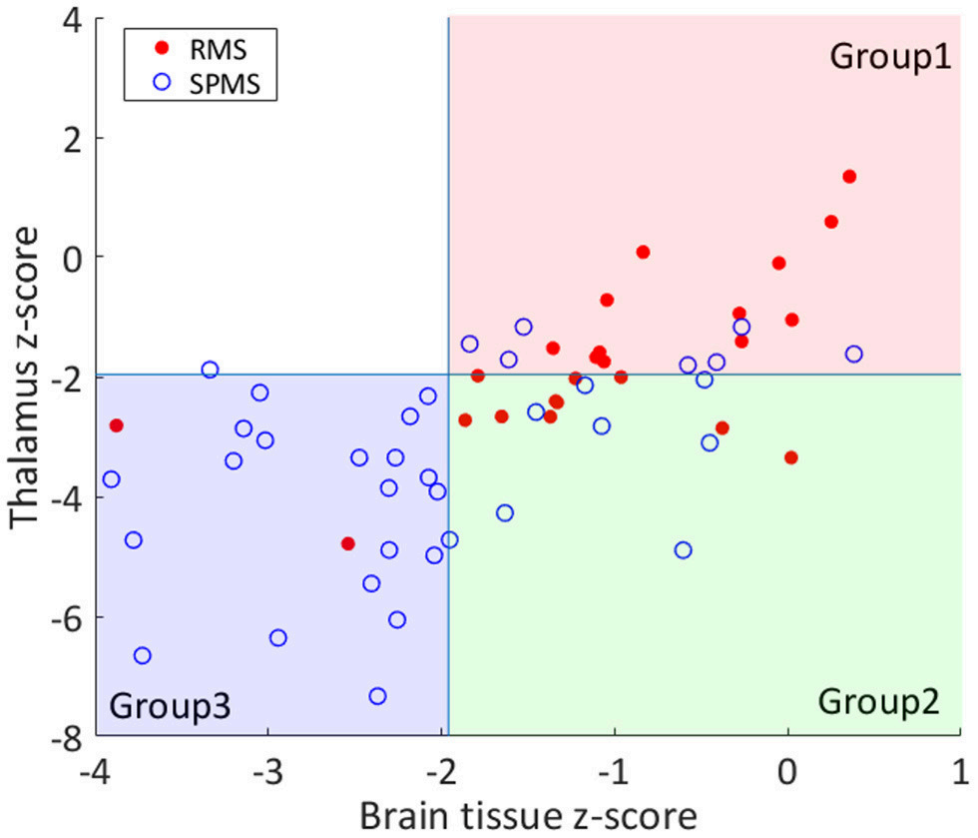
Grouping of the patients based on thalamic and BP atrophy. The RMS and SPMS patients were divided into groups based on thalamus and brain parenchyma (BP) volume *z*-scores. The z-scores were computed by comparing individual brain volumes against healthy controls. A *z*-score cut-off of −1.96 was applied to separate atrophic from normal brain volumes for thalamus and whole BP. RMS patients are marked with filled red circles and SPMS patients are marked with blue rings. Most of the RMS patients fell into Group 1 (both normal BP and thalamus). Group 2 patients had thalamic atrophy without BP atrophy. Most of the SPMS patients had both BP and thalamic atrophy (Group 3). As shown, only 1 patient had BP atrophy without thalamic atrophy (upper left quadrant).

After the comparisons between the RMS and SPMS patient groups, the patients were divided into new subgroups based on thalamic and BP atrophy using a *z*-score cut-off value of −1.96. At the study baseline, 7 SPMS and 12 RMS patients had no brain atrophy, 8 SPMS and 10 RMS patients had isolated thalamic atrophy and 2 RMS and 20 SPMS patients had both BP and thalamic atrophy. Only one SPMS patient had BP atrophy without thalamic atrophy. The patient group that had no thalamic and no BP atrophy was named Group 1 (*n* = 19). The group with thalamic atrophy without BP atrophy was named Group 2 (*n* = 18). NEDA could not be determined for two patients in Group 2 because of missing two-year MRI and EDSS change could not be determined for one patient due to missing two-year EDSS data. The group of patients with both BP and thalamic atrophy were named Group 3 (*n* = 22). The grouping of the patients according to their thalamic and BP atrophy is illustrated in Figure 1. The one SPMS patient that had BP atrophy without thalamic atrophy (Group 4, *n* = 1), was very close to the cut-off value of the patients in Group 3 (thalamus *z*-score −1.88, whole BP −3.34), and one patient fell in between Groups 1 and 2 and could not be categorized in either group.

### Subgroup Comparisons Between the Groups Formed Based on Atrophy Measures

After grouping of the patients according to atrophy measures, between-group differences in EDSS and SDMT at baseline and at 2 years and the achievement of NEDA-3 and NEDA-4 during the follow-up were tested. There was no significant difference in the EDSS, relapses or SDMT points between Groups 1 and 2 at baseline. In Group 3, most of the patients (20/22) were SPMS patients and had higher EDSS, lower SDMT and less relapses compared to patients in Group 1 and 2 and were therefore not included in the between-group outcomes analyses (data not shown).

At the 2-years follow-up, a significant difference was detected between the Groups 1 and 2 in EDSS change. EDSS was same or better at follow-up in 16/19 patients in Group 1, but only in 5/17 patients in Group 2 (*p* = 0.002). NEDA-3 was reached in 11/19 patients in Group 1, but only in 2/17 patients in Group 2 (*p* = 0.012). NEDA-4 was reached in 7 out of the 19 patients in Group 1. Only one of the 16 patients in Group 2 reached NEDA-4 (*p* = 0.047).

There was no significant difference in SDMT scores or relapses between the groups at baseline or at follow-up. SDMT scores had deteriorated during the follow-up in 6/19 patients in Group 1 and in 10/16 patients in Group 2 (*p* = 0.179). In Group 1, 13/19 patients and in Group 2, 13/18 patients had no relapses during the follow-up. A total of 6/19 patients in Group 1 and 5/18 patients in Group 2 had at least 1 relapse during the follow-up (*p* = 1.000). Results are shown in Tables 4, 5.

**Table 4:** Achievement of NEDA-3 and NEDA-4 during the 2-year follow-up in Group 1 and Group 2.

	**NEDA-3**		**NEDA-4**		**Relapses**	
**Group**	**No(n)**	**Yes(*n*)**	**No(*n*)**	**Yes(*n*)**	**>0 (*n*)**	**0 (*n*)**
Group 1^*^	8	11	12	7	6	13
Group 2^**^	14	2	15	1	5	13
*p*-value	**0.012**		**0.047**		1.000	

* Group 1 = Patients with no thalamic or whole BP atrophy.

** Group 2 = Patients with thalamic atrophy and no whole BP atrophy.

Prevalence of NEDA3-No: 63%.

Sensitivity: 64%.

Specificity: 85%.

Positive predictive value: 88%.

Negative predictive value: 58%.

Odds ratio (OR) [95% CI]: 9.6 [1.69, 54.79].

The bold values indicate statistically significant p-values.

**Table 5:** EDSS and SDMT change during the 2-year follow-up in Group 1 and Group 2.

	**EDSS change**		**SDMT change**	
**Group**	**Same/better (*n*)**	**Worse (*n*)**	**Same/better (*n*)**	**Worse (*n*)**
Group 1^*^	16	3	13	6
Group 2^**^	5	12	7	10
*p*-value	**0.002**		0.179	

* Group 1 = Patients with no thalamic or whole BP atrophy.

** Group 2 = Patients with thalamic atrophy and no whole BP atrophy.

Prevalence of EDSS change-Worse: 42%.

Sensitivity: 80%.

Specificity: 76%.

Positive predictive value: 71%.

Negative predictive value: 84%.

Odds ratio (OR) [95% CI]: 12.8 [2.55, 64.37].

The bold values indicate statistically significant p-values.

## Discussion

Thalamus and other deep GM nuclei are among the first GM structures to be affected in MS. Previous studies have suggested that subcortical atrophy might precede whole brain atrophy in MS (6, 14, 28). In this study, only 1/60 MS patients had whole BP atrophy without thalamic atrophy, but thalamic atrophy without whole BP atrophy could be shown in 16/60 patients, indicating that thalamic atrophy is meaningfully clinically measurable before whole brain atrophy. Further, a total of 20 out of the 22 patients who had both whole BP and thalamic atrophy were SPMS patients.

Thalamic atrophy has been shown to correlate with accumulation of disability in patients with MS (8). The unique role of the thalamus in predicting future disability, in comparison to other subcortical structures, has been shown in previous studies (14, 29). Eshaghi et al. (29) found that smaller deep gray matter volume at baseline was associated with increased risk of shorter time to EDSS progression and that thalamus was a better predictor of future disability than other deep gray matter regions. In our study, thalamic atrophy significantly correlated both with worse performance in SDMT test and with higher EDSS score. Patients with isolated thalamus atrophy were at a higher risk for not reaching 2-year NEDA-3 and for EDSS increase than patients with no identified brain atrophy. However, 4 out of the 11 patients with no thalamic or whole brain atrophy at baseline and having NEDA-3 at 2 years, still developed brain atrophy exceeding 0.4% annual atrophy rate in the 2 years follow up (>0.8% brain atrophy at 2 years). This result is in line with the study by Uher et al. (30) suggesting that reaching NEDA-4 at least on platform MS therapies is a hard goal to achieve.

Based on relapse activity, EDSS and SDMT, the RMS patients with isolated thalamus atrophy and no detectable brain atrophy were clinically not distinguishable (*p* > 0.05). Most of the patients falling within the group of isolated thalamus atrophy were treatment naïve RMS patients and they were treated with either IFN or GA during the study. Our results suggest that isolated thalamus atrophy could serve as a subclinical prognostic factor associated with the NEDA-3 outcome measure on the injectable platform therapies. Measuring thalamic and whole brain atrophy could thence help to identify patients with a more severe disease and need for the most effective therapies from early on.

The association of global and regional brain atrophy with disability and the differences between RMS and SPMS patients considering atrophy measures have been reported in several previous studies (3, 29, 31, 32). In line with previous findings, we detected significant differences in total and regional brain volumes between newly diagnosed RRMS patients and SPMS patients. In the early RRMS group, WM lesion volume was smaller, whereas total brain, total cerebral GM, thalamus, hippocampus, cerebellar WM and putamen volumes were greater than in the SPMS group. Differences in volumes were most significant for thalamus and cerebellar WM. SPMS patients have a longer history of the neurodegenerative disease process explaining their progression to greater extent of brain atrophy irrespective of their age. The age difference between the RMS and SPMS patients could not solely explain the observed differences in brain volumes because the brain volumes were normalized for age in the analyses.

Brain volume assessment involves multiple confounding variables, which make its clinical applicability challenging. These include the physiological variables of the patient, such as age, sex, head size, hydration state, menstrual cycle, smoking, alcohol consumption, and comorbidities (e.g., hypertension and diabetes). MRI-related variables include differences between scanners, acquisition protocol, quantification method, position in the scanner, and head motion. MS-related variables include brain lesions, pseudo atrophy, disease duration, inflammation, and drug treatment (33, 34). In this study, we aimed to take into account the confounding variables. We gathered information of comorbidities, education and life style factors. The regional brain volumes were normalized for age, gender and head size. There were no significant differences between the two groups of patients in their comorbidities, alcohol consumption, education status, smoking habits, serum vitamin D levels, or BMI. All of the brain MRIs were acquired with the same Siemens MRI scanner and protocol. Unfortunately, we did not have normative data available from exactly the same scanner model that was used in for the MS patients. However, we analyzed our data using normative data from more than 400 cases from 8 different Siemens scanner models from the OASIS data base (27) showing relatively consistent results: the 95 % confidence interval of the average thalamus volume for each scanner contains the average thalamus volume computed for all eight scanners. Because of this, we think that using normative data from Siemens 3T scanners is a feasible compromise. If enough normative data were available, it would be ideal to compute the z-scores using data from exactly the same scanner model. In clinical practice, it may also be challenging to always scan the patient in the same scanner.

In line with Raji et al. (14), we found that it is possible to detect isolated thalamus atrophy by MRI-based brain volumetry at a single time point. A single time point predictive MRI brain volume measure could help implementing brain volume measurement into the clinical practice, since measuring brain volume changes between time points is technically challenging outside clinical trial settings (2). Segmentation based methods for brain volume assessment, such as SIENAX, are not designed for the assessment of volume change between time points.

The discussed challenges in brain volume assessment are among the reasons, why brain atrophy is not routinely measured in MS clinical practice. However, being relatively easy to carry out, measuring thalamic atrophy at a single time point could be applicable in clinical practice.

## Conclusion

Our study supports the usefulness of a single measurement of thalamic and whole brain atrophy in identification of patients at risk of disability progression and not reaching NEDA-3 with platform injectable therapies. The results of our study have to be considered as pilot ones, since the number of patients in our study was rather small and the follow-up time of 2 years is short for detection of confirmed disability progression. Further studies with larger cohorts of patients, longer follow and possibly complemented by addition of other prognostic markers such as neurofilament will hopefully be seen in near future.

## Ethics Statement

The study was performed in accordance with the ethical standards laid down in the 1964 Declaration of Helsinki and its later amendments. All persons included in the study gave their informed consent prior to their inclusion in the study.

## Author Contributions

KH, MS-H, and JL contributed conception and design of the study. KH, MS-H, and MV organized the database. MV performed the statistical analysis. KH wrote the first draft of the manuscript. MV wrote the statistics section of the manuscript and JOK and TP the MRI acquisition section of the manuscript. All authors contributed to manuscript revision, read and approved the submitted version.

### Conflict of Interest Statement

MS-H has received honoraria for lectures, advisory boards, or for serving as an investigator for clinical trials from Bayer, Biogen, Eisai, Novartis, Orion, Roche, Sanofi-Genzyme, Teva, and UCB. JL is CEO and JuK are employees and shareholders at Combinostics. TP has received a lecture fee from Roche. JR serves as a consultant neurologist for CRST (Clinical Research Services Turku). MV has been an employee at StellarQ since October 2018. The remaining authors declare that the research was conducted in the absence of any commercial or financial relationships that could be construed as a potential conflict of interest.
