# Supplementary material for: Thalamic Atrophy Without Whole Brain Atrophy Is Associated With Absence of 2-Year NEDA in Multiple Sclerosis
Source: Front Neurol. 2019 May 3;10:459. doi: 10.3389/fneur.2019.00459 (PMC6509198; doi:10.3389/fneur.2019.00459)
Supplement: Supplementary file 1 [file Table_1.DOCX]

Supplemental Table 1. Disease modifying therapies in the RMS and SPMS patients______________

| Patient | Start | Stop | Drug | Drug name | Possible reason to stop |
| --- | --- | --- | --- | --- | --- |
| RRMS01 | 1.10.2014 | 10.6.2014 | 1 | Avonex | Inefficient |
| RRMS01 | 16.6.2014 |  | 7 | Tysabri |  |
| RRMS02 | 13.9.2013 |  | 1 | Avonex |  |
| RRMS03 | 9.9.2013 |  | 1 | Avonex |  |
| RRMS04 | 16.9.2013 |  | 3 | Copaxone |  |
| RRMS05 | 14.1.2014 |  | 1 | Avonex |  |
| RRMS05 | 19.9.2013 | 2014/01 | 6 | Rebif | Adverse effects |
| RRMS05 | 19.9.2013 | 12.12.2013 | 6 | Rebif | Adverse effects |
| RRMS06 | 12.9.2013 |  | 1 | Avonex |  |
| RRMS06 | 19.8.2015 |  | 37 | Tecfidera |  |
| RRMS07 | 10.10.2013 | 14.4.2015 | 3 | Copaxone | Inefficient |
| RRMS07 | 16.4.2015 |  | 5 | Gilenya |  |
| RRMS08 | 1.10.2013 | 2015/01 | 1 | Avonex | Pregnancy |
| RRMS09 | 2015/06 |  | 3 | Copaxone |  |
| RRMS10 | 20.1.2014 | 2.5.2014 | 3 | Copaxone | Inefficient |
| RRMS10 | 7.10.2013 | 8.12.2013 | 6 | Rebif | Adverse effects |
| RRMS10 | 21.5.2014 |  | 7 | Tysabri |  |
| RRMS11 | 23.10.2013 |  | 3 | Copaxone |  |
| RRMS11 | 24.5.2016 |  | 3 | Copaxone |  |
| RRMS12 | 21.1.2014 | 18.10.2016 | 3 | Copaxone | Inefficient |
| RRMS12 | 28.10.2013 | 9.1.2014 | 1 | Avonex | Adverse effects |
| RRMS12 | 1.7.2016 |  | 37 | Tecfidera |  |
| RRMS12 | 18.10.2016 |  | 37 | Tecfidera |  |
| RRMS13 | 20.11.2013 | 1.9.2016 | 6 | Rebif | Inefficient |
| RRMS13 | 8.9.2016 |  | 5 | Gilenya |  |
| RRMS14 | 12.12.2013 | 31.1.2016 | 6 | Rebif | Compliance |
| RRMS14 | 29.2.2016 |  | 37 | Tecfidera |  |
| RRMS15 | 2.12.2013 | 2.4.2015 | 1 | Avonex | Pregnancy |
| RRMS15 | 7.3.2016 |  | 1 | Avonex |  |
| RRMS16 | 10.9.2013 |  | 6 | Rebif |  |
| RRMS17 | 31.1.2014 | 3.3.2016 | 3 | Copaxone | Other |
| RRMS17 | 6.2.2017 |  | 3 | Copaxone |  |
| RRMS18 | 7.2.2014 | 2015 | 6 | Rebif | Inefficient |
| RRMS18 | 2014/08 | 2015/06 | 3 | Copaxone | Inefficient |
| RRMS18 | 24.6.2015 | 22.1.2016 | 5 | Gilenya | Inefficient |
| RRMS18 | 15.3.2016 |  | 7 | Tysabri |  |
| RRMS19 | 11.3.2014 | 2015/07 | 6 | Rebif | Inefficient |
| RRMS19 | 3.8.2015 |  | 5 | Gilenya |  |
| RRMS19 | 3.8.2015 |  | 5 | Gilenya |  |
| RRMS20 | 25.4.2014 |  | 6 | Rebif |  |
| RRMS21 | 13.5.2014 | 12.7.2016 | 6 | Rebif | Inefficient |
| RRMS21 | 1.8.2016 |  | 5 | Gilenya |  |
| RRMS22 | 30.5.2014 |  | 6 | Rebif |  |
| RRMS23 | 27.6.2014 | 6.11.2015 | 1 | Avonex | Pregnancy |
| RRMS24 | 24.7.2014 |  | 1 | Avonex |  |
| SPMS01 | 2002/02 | 29.3.2007 | 2 | Betaferon | Antibodies |
| SPMS02 | 2005/06 | 1.4.2016 | 2 | Betaferon | Compliance |
| SPMS02 | 7.6.2005 | 31.3.2016 | 2 | Betaferon | Other |
| SPMS02 | 1.4.2016 |  | 38 | Aubagio |  |
| SPMS03 | 25.11.2005 | 13.4.2007 | 6 | Rebif | Antibodies |
| SPMS03 | 14.4.2007 | 26.4.2016 | 3 | Copaxone | Compliance |
| SPMS03 | 2007 | 26.4.2016 | 3 | Copaxone | Compliance |
| SPMS03 | 26.4.2016 |  | 3 | Copaxone |  |
| SPMS04 | 15.2.2003 |  | 3 | Copaxone |  |
| SPMS04 | 1994/02 | 5.5.2003 | 6 | Rebif | Antibodies |
| SPMS05 | 2001/09 | 2002/01 | 6 | Rebif | Adverse effects |
| SPMS05 | 2002/02 | 2005/04 | 3 | Copaxone | Disease nature change |
| SPMS06 | 1994/04 |  | 6 | Rebif |  |
| SPMS06 | 13.2.2002 |  | 3 | Copaxone |  |
| SPMS06 | 1994/09 | 2002/02 | 6 | Rebif | Compliance |
| SPMS06 | 2002/03 | 2012/07 | 3 | Copaxone | Compliance |
| SPMS06 | 20.2.2013 |  | 3 | Copaxone |  |
| SPMS08 | 2000/01 | 2004/10 | 6 | Rebif | Antibodies |
| SPMS08 | 2004/11 | 2005/04 | 3 | Copaxone | Inefficient |
| SPMS08 | 6.7.2016 |  | 38 | Aubagio |  |
| SPMS09 | 1998/11 | 2003/11 | 2 | Betaferon | Antibodies |
| SPMS09 | 2003/11 | 2005/05 | 1 | Avonex | Antibodies |
| SPMS09 | 1.6.2005 | 17.8.2010 | 3 | Copaxone | Disease nature change |
| SPMS10 | 8.12.2014 |  | 38 | Aubagio |  |
| SPMS10 | 2009/06 | 28.10.2014 | 2 | Betaferon | Compliance |
| SPMS11 | 4.3.2015 |  | 1 | Avonex |  |
| SPMS14 | 7.10.2011 | 2014/08 | 1 | Avonex | Disease nature change |
| SPMS16 | 2005 |  | 6 | Rebif |  |
| SPMS17 | 2.3.2010 | 7.10.2013 | 3 | Copaxone | Disease nature change, compliance |
| SPMS17 | 1998 | 10.11.2009 | 6 | Rebif | Adverse effects |
| SPMS18 | 19.3.2002 | 12.9.2002 | 3 | Copaxone | Other |
| SPMS18 | 1994/09 | 2000/01 | 6 | Rebif | Other |
| SPMS18 | 2001/03 | 19.3.2002 | 6 | Rebif | Compliance |
| SPMS18 | 1992 | 1997 | 6 | Rebif | Other |
| SPMS19 | 1997/10 | 6.8.1999 | 1 | Avonex | Inefficient |
| SPMS19 | 6.8.2002 | 25.2.2009 | 6 | Rebif | Disease nature change |
| SPMS21 | 13.8.2003 |  | 3 | Copaxone |  |
| SPMS22 | 20.1.2003 | 26.10.2010 | 2 | Betaferon | Adverse effects, other |
| SPMS22 | 5.5.2011 | 4.9.2011 | 3 | Copaxone | Adverse effects |
| SPMS22 | 2015/04 | 8.4.2016 | 37 | Tecfidera | Adverse effects |
| SPMS22 | 1.1.2016 |  | 38 | Aubagio |  |
| SPMS23 | 14.2.2007 | 2010/06 | 3 | Copaxone | Disease nature change |
| SPMS23 | 2001 | 14.2.2007 | 6 | Rebif | Inefficient |
| SPMS24 | 14.7.2003 | 2010/03 | 6 | Rebif | Disease nature change |
| SPMS25 | 3.4.1997 | 2011 | 2 | Betaferon | Disease nature change |
| SPMS26 | 1993 | 1999 | 6 | Rebif | Adverse effects |
| SPMS26 | 1992/01 | 1999/01 | 2 | Betaferon | Adverse effects |
| SPMS27 | 2005/06 | 5.3.2007 | 2 | Betaferon | Inefficient |
| SPMS27 | 5.3.2007 | 12.4.2012 | 3 | Copaxone | Disease nature change |
| SPMS27 | 3.4.2013 | 1.4.2015 | 3 | Copaxone | Other |
| SPMS28 | 11.10.2016 |  | 37 | Tecfidera |  |
| SPMS29 | 22.12.2016 | 23.12.2016 | 38 | Aubagio | Other |
| SPMS31 | 2006/03 | 2014/06 | 3 | Copaxone | Compliance |
| SPMS32 | 2005/01 | 10.6.2010 | 1 | Avonex | Disease nature change |
| SPMS34 | 1.12.2005 | 2007 | 3 | Copaxone | Other |
| SPMS35 | 19.10.2009 | 24.1.2015 | 3 | Copaxone | Adverse effects |
| SPMS36 | 2001/01 | 2004/11 | 6 | Rebif | Antibodies |
| SPMS36 | 2005/06 | 8.12.2005 | 3 | Copaxone | Adverse effects |

All patients in the RMS group were treatment naive at the study onset. In the SPMS group also previous medication history is shown. First patient enrolled in March 2014 and last patient in January 2015.
