# Supplementary material for: Thalamic Atrophy Without Whole Brain Atrophy Is Associated With Absence of 2-Year NEDA in Multiple Sclerosis
Source: Front Neurol. 2019 May 3;10:459. doi: 10.3389/fneur.2019.00459 (PMC6509198; doi:10.3389/fneur.2019.00459)
Supplement: Supplementary file 2 [file Table_2.DOCX]

Supplemental Table 2. Numbers of T2-lesions and Gadolinium-enhancing lesions in the RMS and SPMS patients at study baseline and at 2 years

| Patient | Baseline_gadolinium | Control_gadolinium | Change_gado | Baseline_T2Total | Control_T2Total |
| --- | --- | --- | --- | --- | --- |
| RRMS01 | 10-20 | 0 | Better | 21-40 | 21-40 |
| RRMS02 | 0 | 0 | Same | 10-20 | 10-20 |
| RRMS03 | 0 | 0 | Same | 10-20 | 10-20 |
| RRMS04 | 0 | 0 | Same | 10-20 | 10-20 |
| RRMS05 | 0 | 0 | Same | 10-20 | 10-20 |
| RRMS06 | 0 | 0 | Same | 10-20 | 10-20 |
| RRMS07 | 0 | 0 | Same | 10-20 | 10-20 |
| RRMS08 | 0 | 0 | Same | 21-40 | 21-40 |
| RRMS09 | 0 | 0 | Same | 21-40 | 21-40 |
| RRMS10 | 0 | 0 | Same | 21-40 | 21-40 |
| RRMS11 | 0 | 0 | Same | 10-20 | 10-20 |
| RRMS12 | 0 | 0 | Same | 7 | 7 |
| RRMS13 | 1 | 0 | Better | >40 | >40 |
| RRMS14 | 0 | 0 | Same | 21-40 | 21-40 |
| RRMS15 | 0 | 1 | Worse | 9 | 10-20 |
| RRMS16 | 0 | 0 | Same | 10-20 | 10-20 |
| RRMS17 | 0 | 0 | Same | 10-20 | 10-20 |
| RRMS18 | 0 | 0 | Same | 10-20 | 10-20 |
| RRMS19 | 0 | 0 | Same | >40 | >40 |
| RRMS20 | 1 | 0 | Better | 10-20 | 10-20 |
| RRMS21 | 0 | 0 | Same | 3 | 3 |
| RRMS22 | 1 |  |  | >40 |  |
| RRMS23 | 0 | 0 | Same | 5 | 5 |
| RRMS24 | 0 | 0 | Same | 10-20 | 10-20 |
| SPMS01 | 0 | 0 | Same | 21-40 | 21-40 |
| SPMS02 | 0 | 0 | Same | 21-40 | 21-40 |
| SPMS03 | 0 | 0 | Same | >40 | >40 |
| SPMS04 | 0 | 0 | Same | 21-40 | 21-40 |
| SPMS05 | 0 | 4 | Worse | >40 | >40 |
| SPMS06 | 0 | 0 | Same | >40 | >40 |
| SPMS07 | 0 | 0 | Same | 10-20 | 10-20 |
| SPMS08 | 2 | 2 | Same | 21-40 | 21-40 |
| SPMS09 | 0 |  |  | 21-40 |  |
| SPMS10 | 0 | 0 | Same | >40 | >40 |
| SPMS11 | 0 | 0 | Same |  |  |
| SPMS12 | 0 | 0 | Same | >40 | >40 |
| SPMS13 | 0 | 0 | Same | 21-40 | >40 |
| SPMS14 | 0 | 0 | Same | >40 | >40 |
| SPMS15 | 0 | 0 | Same | >40 | >40 |
| SPMS16 | 0 | 0 | Same | 21-40 | 21-40 |
| SPMS17 | 0 | 0 | Same | >40 | >40 |
| SPMS18 | 0 | 0 | Same | >40 | >40 |
| SPMS19 | 0 | 0 | Same | >40 | >40 |
| SPMS20 | 0 | 0 | Same | >40 | >40 |
| SPMS21 | 0 | 0 | Same | >40 | >40 |
| SPMS22 | 0 | 0 | Same | >40 | >40 |
| SPMS23 | 0 | 0 | Same | 21-40 | 21-40 |
| SPMS24 | 2 | 0 | Better | >40 | >40 |
| SPMS25 | 0 | 0 | Same |  |  |
| SPMS26 | 0 | 0 | Same | 21-40 | 21-40 |
| SPMS27 | 0 | 0 | Same | >40 | >40 |
| SPMS28 | 0 | 0 | Same | 21-40 | 21-40 |
| SPMS29 | 0 | 0 | Same | 21-40 | 21-40 |
| SPMS30 | 0 | 0 | Same | >40 | >40 |
| SPMS31 | 0 | 0 | Same | 10-20 | 10-20 |
| SPMS32 | 0 | 0 | Same | >40 | >40 |
| SPMS33 | 0 | 1 | Worse | >40 | >40 |
| SPMS34 | 0 | 3 | Worse | >40 | >40 |
| SPMS35 | 0 | 0 | Same | 21-40 | 21-40 |
| SPMS36 | 0 | 0 | Same | >40 | >40 |
